# Dexmedetomidine Induced Polyuria in the Intensive Care Unit

**DOI:** 10.1155/2021/8850116

**Published:** 2021-02-20

**Authors:** Mohammed M. Uddin, Joseph Sebastian, Muhammad Usama, Fazal I. Raziq, Ghulam Saydain, Noreen F. Rossi

**Affiliations:** ^1^Internal Medicine, Wayne State University/Detroit Medical Center, 4201 St Antoine, Detroit, MI 48201, USA; ^2^Internal Medicine, Michigan State University/Sparrow Hospital, 1215 E Michigan Ave, Lansing, MI 48912, USA; ^3^Pulmonary Criticasl Care & Sleep Division, Wayne State University/Detroit Medical Center, 4201 St Antoine, Detroit, MI 48201, USA; ^4^Division of Nephrology, Wayne State University/Detroit Medical Center, 4201 St Antoine, Detroit, MI 48201, USA

## Abstract

Dexmedetomidine is an *α*2-adrenergic used as an adjunct therapy for sedation in the intensive care unit. While it is known to cause polyuria exclusively in perioperative conditions, not many cases are known in the intensive care unit, thus making the diagnosis challenging. We present the case of a 61-year-old male who had developed polyuria secondary to central diabetes insipidus after receiving dexmedetomidine intravenous infusion in the medical ICU. Increased awareness of this uncommon side effect of dexmedetomidine will help clinicians recognize and address it early.

## 1. Introduction

The use of dexmedetomidine is a common practice in the medical intensive care units (ICU) to minimize delirium and to serve as an adjunctive sedative therapy in intubated patients. Morelli et al. demonstrated that dexmedetomidine is associated with decreased norepinephrine requirements in septic shock patients as compared with propofol [1]. While hypotension and bradycardia [2] are known side effects, the occurrence of central diabetes insipidus (DI) due to dexmedetomidine is a rare phenomenon and not well documented in literature [3, 4]. Rapid onset of dehydration due to fluid loss and electrolyte imbalance may further worsen hypotension and cause complications in already critically ill patients. There is a need for widespread awareness of this rare but potentially serious side effect of dexmedetomidine among critical care physicians, so that this is included in the differential diagnosis early on in cases of polyuria among critically ill patients. We report a case of polyuria in a complex critically ill patient where dexmedetomidine was not initially suspected as a causative agent.

## 2. Case Report

A 61-year-old male with a past medical history of advanced stage COPD, heart failure with reduced ejection fraction, was admitted to the medical ICU with acute on chronic hypercapnic respiratory failure secondary to exacerbation of his chronic obstructive pulmonary disease requiring ventilatory support. He was treated with steroids and azithromycin. His kidney function stayed stable with average urine output of around 2 liters per 24 hours for the first 7 days of hospitalization. On the eighth day of hospitalization, he developed abrupt onset polyuria with an increase in urine output from 70-80 cc/hr. to 200-225 cc/hr., totaling approximately 5.4 L over the next 24 hr. Urine osmolality was 193 mOsm/kg H2O, while his random urine sodium also increased from 23 to 179 mmol per liter urine.

The cause was not immediately apparent. His medications included aspirin, albuterol, isosorbide mononitrate, amiodarone, lisinopril, and metoprolol. CT scan of the head was negative for any intracranial processes ruling out a pituitary lesion as the cause of central DI. The patient was not on any medication known to cause nephrogenic DI and serum electrolytes were within normal limits. The patient was euvolemic and had normal serum glucose, BUN, and other organic acid levels ruling out osmotic diuresis as the cause of polyuria. Renal ultrasound was negative for any obstruction. Pertinent labs included BUN 16 mg/dL, creatinine 0.65 mg/dL, glucose 130 mg/dL, sodium 134 mmol/L, potassium 3.6 mmol/L, and calcium 8.8 mg/dL. Urine electrolytes were sodium 179 mmol/L, potassium 14 mmol/L, chloride 25 mmol/L, creatinine 37 mg/dL, and lithium level <0.1 mmol/L.

A thorough chart review identified that the patient had received dexmedetomidine due to worsening delirium at a rate of 0.2 mcg/kg/hr for a brief period, immediately before the abrupt onset of polyuria. No other instigating cause was identified; we suspected central DI secondary to dexmedetomidine. A DDAVP stimulation test was planned as a therapeutic challenge for central DI, but 48 hours after the cessation of dexmedetomidine, the urine output spontaneously decreased to 50-100 cc/hr and urine osmolality increased to 306 mOsm/kgH2O so the test was not done. His serum osmolality also normalized after that. The patient was not rechallenged with dexmedetomidine. At no time did the patient develop hypernatremia due to strict replacement of carefully calculated water losses.

In the light of extensive negative workup and temporal relationship of polyuria with dexmedetomidine infusion and resolution of the polyuria with drug abstinence, we concluded that the polyuria was secondary to transient central Diabetes Insipidus secondary to dexmedetomidine infusion. The Naranjo Score for dexmedetomidine induced polyuria for this patient was 8, thereby putting the medication in the probable likelihood of causing the adverse reaction of polyuria [5].

## 3. Discussion

Polyuria is defined as a urinary output of greater than 3 L/day, the causes of which can be divided into solute (osmotic) and water diuresis. Solute diuresis most commonly occurs secondary to glycosuria, whereas water diuresis occurs either due to a defect in antidiuretic hormone (ADH) production or due to decreased renal responsiveness to ADH.

In this case, a thorough review was negative for osmotic diuresis secondary to hyperglycemia, azotemia, or intravenous volume expansion. There was no evidence of neuroendocrine disorders such as head trauma as evidenced by the CT scan, acute renal pathologies such as obstruction, or recovery from acute kidney injury or the use of diuretics that could trigger polyuria. The low urine osmolality could not be attributed to hypokalemia, hypercalcemia, or lithium use that is associated with nephrogenic diabetes insipidus.

**Figure 1:**
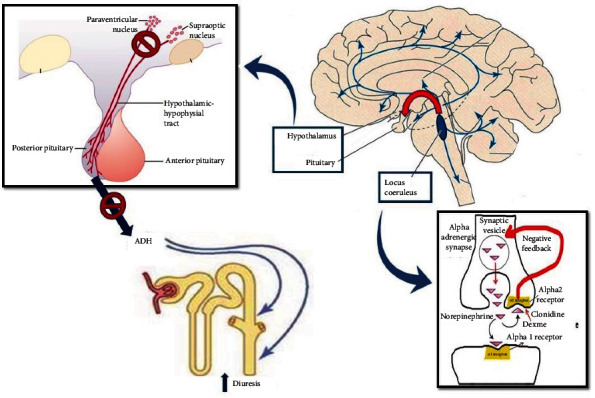
Neural pathways involved in dexmedetomidine-induced central diabetes insipidus.

To our knowledge, only one other case in a medical intensive care unit has been published in abstract form [3]. Polyuria associated with dexmedetomidine, a highly selective *α*2-adrenoceptor agonist that works on the locus coeruleus, has been reported in case report form, almost exclusively in perioperative conditions [6–8]. Lesions of the locus coeruleus have been associated with decreased secretion of ADH [8, 9] thereby resulting in transient central diabetes insipidus. Early work by Sawchenko and Swanson [10] and others [11] demonstrated noradrenergic pathways from the locus coeruleus to the paraventricular and supraoptic nuclei (Figure 1). These pathways have been shown to modulate ADH release from the posterior pituitary into the peripheral circulation particularly under conditions of physiologic stress [12].

Previously reported cases occurred primarily in the operative setting under anesthesia. They also showed a rapid resolution of polyuria once dexmedetomidine was withheld [6, 7]. Our case is unique in that it occurred within the medical ICU setting and that the effect persisted for 48 hr after stopping dexmedetomidine. The half-life of dexmedetomidine is only 3.7 hr; however, its metabolite has a half-life of 9.7 hr [2]. At least two possibilities present themselves. Either the concurrent administration of drugs metabolized via several CYP450 enzymes prolonged the half-life or the H-3 metabolite or another breakdown product may have an impact on ADH secretion manifesting as polyuria. Our case is distinct in that it highlights that even without a loading dose and a relatively low dose infusion (0.2 mcg/kg/hr) of dexmedetomidine with short duration had induced polyuria that persisted well beyond the time limit in the previously reported cases, suggesting that the side effects are likely to be dose independent.

## 4. Conclusion

Increased awareness of this uncommon but significant side effect of dexmedetomidine will promote providers to include it in the differential diagnoses when assessing for polyuria in the intensive care unit while, at the same time, promoting careful monitoring of concurrent medications, underlying liver disease, and urine output, particularly in susceptible patients.

## Data Availability

None.
